# The Impact of Biowaste Composition and Activated Carbon Structure on the Electrochemical Performance of Supercapacitors

**DOI:** 10.3390/molecules29215029

**Published:** 2024-10-24

**Authors:** Alisher Abdisattar, Meir Yerdauletov, Mukhtar Yeleuov, Filipp Napolskiy, Aleksey Merkulov, Anna Rudnykh, Kuanysh Nazarov, Murat Kenessarin, Ayazhan Zhomartova, Victor Krivchenko

**Affiliations:** 1 Bes Saiman Group, 050057 Almaty, Kazakhstan; 2 Satbayev University, 050000 Almaty, Kazakhstan; 3 Engineering and Science Hub, 050004 Almaty, Kazakhstan; 4 Institute of Nuclear Physics, 050032 Almaty, Kazakhstan; k.nazarov@inp.kz (K.N.);; 5 Joint Institute for Nuclear Research, 141980 Dubna, Russia; 6 Dubna State University, 141980 Dubna, Russia

**Keywords:** biomass composition, wheat bran, wheat straw, barley straw, activated carbon, FTIR spectra, supercapacitor

## Abstract

The increasing demand for sustainable and efficient energy storage materials has led to significant research into utilizing waste biomass for producing activated carbons. This study investigates the impact of the structural properties of activated carbons derived from various lignocellulosic biomasses—barley straw, wheat straw, and wheat bran—on the electrochemical performance of supercapacitors. The Fourier Transform Infrared (FTIR) spectroscopy analysis reveals the presence of key functional groups and their transformations during carbonization and activation processes. The Raman spectra provide detailed insights into the structural features and defects in the carbon materials. The electrochemical tests indicate that the activated carbon’s specific capacitance and energy density are influenced by the biomass source. It is shown that the wheat-bran-based electrodes exhibit the highest performance. This research demonstrates the potential of waste-biomass-derived activated carbons as high-performance materials for energy storage applications, contributing to sustainable and efficient supercapacitor development.

## 1. Introduction

The increasing demand for sustainable and efficient materials has driven significant research into the utilization of waste biomass as a renewable source for producing activated carbons for high-power energy storage systems such as supercapacitors and Li-ion capacitors [1,2]. Waste biomass, a byproduct of various agricultural and industrial processes, offers an abundant and low-cost precursor for activated carbon production. This approach not only addresses waste management challenges but also provides a sustainable pathway for creating high-value materials with diverse applications [3,4].

Activated carbons are renowned for their high surface area, porosity, and adsorption capacitance, making them ideal candidates for environmental and energy storage applications, particularly in the development of electric double-layer capacitors (i.e., supercapacitors) [2,5]. These energy storage devices require materials with high electrical conductivity, electrochemical stability, and a large surface area to achieve high energy and power density [6,7]. The activated carbons derived from waste biomass possess these characteristics, making them promising materials for supercapacitor electrodes.

The composition of lignocellulosic biomass significantly influences the development of pores in activated carbon [8]. Cellulose primarily contributes to the formation of micropores, while lignin helps maintain structural integrity as well as contributes to specific surface area development [9]. The activation process, whether physical or chemical, further refines the pore structure, enhancing the overall adsorption capacitance and effectiveness of the activated carbon. 

Traditional wet chemical methods using two-step sulfuric acid hydrolysis for biomass composition analysis are time-consuming and labor-intensive and do not provide structural information, limiting their suitability for industrial applications or large-scale energy storage systems. In contrast, infrared techniques offer rapid, cost-effective analysis that is non-destructive and have shown promising results [10]. 

FTIR spectroscopy, a robust analytical technique, provides detailed information on the chemical structure and functional groups present in materials [11,12]. It has been successfully utilized to analyze the composition of lignocellulosic biomass. By analyzing the FTIR spectra of waste biomasses as well as carbonized and activated carbons, we can identify the key functional groups and compositional changes that occur during the activation process. This spectral analysis is essential for understanding how the initial composition of the biomass influences the properties of activated carbons and, ultimately, their performance as supercapacitor electrodes.

In this research, we systematically investigated the FTIR spectra of various waste biomasses and their corresponding carbonized and activated carbons. We aimed to correlate the spectral data with the electrochemical properties of the activated carbons to provide a comprehensive understanding of the relationship between the initial biomass composition, the activation-induced changes, and the electrode performance.

In this work, we investigated the structural and electrochemical characteristics of activated carbons synthesized from barley straw (BS), wheat straw (WhS), and wheat bran (WhB). The resulting activated carbons had a specific surface area of 1866 to 2036 m^2^/g.

The study of electrochemical characteristics revealed that the activated carbon synthesized from wheat bran had the highest specific capacitance, reaching 143 F/g at 0.1 A/g.

At the same time, all samples demonstrated high cyclability, which was expressed by a retention of approximately 94% of the initial capacitance after 6000 cycles at a current density of 1 A/g. 

This study not only enhances the fundamental knowledge of biomass-derived activated carbons but also offers practical insights for developing high-performance supercapacitor electrodes from sustainable resources.

## 2. Results and Discussion

### 2.1. Characterization

**Figure 1:**
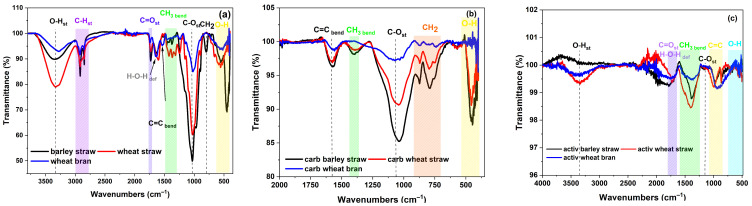
FTIR spectra and composition of functional groups of (**a**) original biomass; (**b**) carbonized biomass; (**c**) activated biomass.

The Fourier Transform Infrared (FTIR) spectroscopy analysis of the biomass samples revealed characteristic peaks, indicating the presence of various functional groups (Figure 1). The b broad peak at 3330 cm^−1^ indicates O-H stretching vibration (Figure 1a). This peak is characteristic of hydroxyl groups, which are prevalent in the cellulose, hemicellulose, and lignin components of biomass [13]. The presence of this peak suggests a significant amount of hydrogen bonding, which is typical in polysaccharides and water molecules within biomass.

The absorption bands observed in the range of 3000–2780 cm^−1^ correspond to the C-H stretching vibrations of aliphatic hydrocarbons. These bands indicate the presence of methylene (CH2) and methyl (CH3) groups, which are common in lignocellulosic materials [14]. The distinct peak at 1727 cm^−1^ is associated with the C=O stretching vibration of carbonyl groups [15]. This peak is typically attributed to esters, aldehydes, and ketones, suggesting the presence of hemicellulose and lignin derivatives in biomass.

The peak at 1640 cm^−1^ is attributed to H-O-H deformation vibration. This peak is often associated with absorbed water and indicates the presence of moisture in the biomass sample [15]. The peaks in the range of 1500–1280 cm^−1^ are assigned to the bending vibrations of CH₃ groups. These peaks suggest the presence of methyl groups, which are part of the lignin and hemicellulose structures in biomass.

The peak at 1030 cm^−1^ indicates C-O stretching vibration. This peak is commonly associated with the C-O-C linkages in cellulose and hemicellulose, confirming the polysaccharide nature of the biomass [9].

As illustrated in Figure 1a, there were no qualitative differences among the various lignocellulosic biomasses; the distinctions were solely in the quantitative ratios of their components. Following carbonization (heat treatment up to 550 °C), the functional groups in the range of 1600–3500 cm^−1^ and the H-O-H deformation vibration at 1640 cm^−1^, associated with absorbed water, completely disappeared due to the volatilization of these substances. Additionally, the intensity of the remaining peaks significantly decreased, indicating the decomposition of the hemicellulose and its associated functional groups, as well as the partial decomposition of the cellulose and lignin.

Notably, a C=C peak representing aromatic rings emerged after carbonization (Figure 1b) at 1540 cm^−1^, likely formed due to the loss of functional groups. It was interesting to observe that the intensity of this peak was higher for barley and wheat straw, which initially exhibited more intense functional groups that volatilized during heat treatment (1600–3500 cm^−1^) compared to wheat bran. This suggests that the more functional groups are lost, the more aromatic rings are formed. Consequently, the remaining functional groups play a crucial role in influencing the structure and properties of activated carbon during further activation of the carbonized mass. The yield of the carbonaceous products after the carbonization process for all the biomass samples was 29–30%.

Of particular interest was the peak at 1500–1280 cm^−1^, corresponding to the CH₃ group, the intensity of which did not significantly decrease after both carbonization and activation (Figure 1). Methyl groups are exceptionally stable, comprising a central carbon atom connected to three hydrogen atoms, and generally remain unreactive even when exposed to strong acids or bases [16]. The intensity of this group remained higher for barley and wheat straw after carbonization and activation, suggesting that these biomasses exhibited the smallest total pore volume in the activated state (Table 1). It appeared that these stable groups blocked the pores during the activation process, as they did not evaporate or decompose at high temperatures or in the presence of KOH.

The FTIR spectra of the activated carbons (Figure 1c) show significantly fewer bands, indicating that most functional groups decomposed during carbonization and activation. This decomposition process is crucial for understanding the structural transformations and the resultant properties of the activated carbons. The yield of the activated carbons after the activation process was 44.7% for wheat bran, 41.75% for barley straw, and 49.5% for wheat straw.

**Figure 2:**
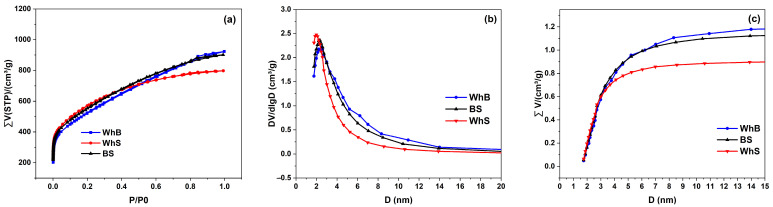
(**a**) Nitrogen adsorption/desorption isotherms, (**b**) differential PSDs, and (**c**) cumulative PSDs for the activated biomasses.

The nitrogen adsorption/desorption isotherm of the samples exhibited a Type Ib isotherm with an H4 hysteresis loop (Figure 2a), indicating the presence of wider micropores and narrow mesopores with high surface area and slit shape [17,18]. The BET surface area and the total pore volume for activated carbons are represented in Table 1.

The differential pore size distribution showed a peak at 2–2.4 nm, confirming the dominance of mesopores in this range (Figure 2b). The cumulative pore size distribution demonstrated a steady increase in pore volume with increasing pore diameter, with a significant rise between 1.7 nm and 3 nm, aligning with the micro- and mesoporous nature of the material (Figure 2c). The cumulative pore volume reached a plateau at around 5 nm, signifying that most of the pore volume was due to mesopores. The consistent pore size distribution across all samples indicated that the activation process effectively generated micropores in the 2.2 nm range, which is beneficial for various adsorption and filtration applications.

Comparing the specific surface area of the activated carbons (Table 1) and the intensity of the peaks at 1030 cm^−1^ corresponding to C-O stretching vibrations (Figure 1b), it was evident that a higher presence of this functional group correlated with a greater specific surface area. This is consistent with findings by Catalina et al. [9], who demonstrated that cellulose possesses the largest specific surface area.

**Figure 3:**
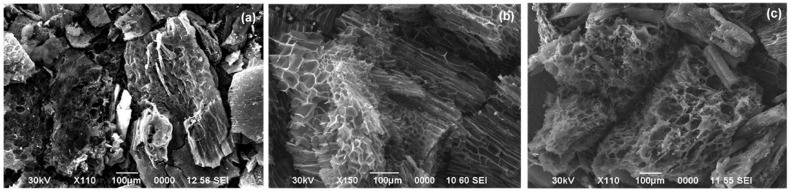
SEM images of activated carbon derived from (**a**) wheat bran; (**b**) wheat straw; (**c**) barley straw.

The wheat bran activated carbon exhibited a layered structure with both micro- and macropores (Figure 3a). The SEM images showed a mix of sheet-like formations and irregularly shaped particles. The particle size varied in a range of few hundred micrometers. The SEM images of wheat straw (Figure 3b) activated carbon revealed a heterogeneous porosity with a mix of micropores and macropores. The surface texture was rough, and remnants of the straw’s cellular structure were evident. The activated carbon derived from barley straw (Figure 3c) displayed a porous network with visible vascular bundles. The surface was rough with notable cracks and fissures, indicative of the breakdown of the straw’s cellular structure. The particles were predominantly elongated and ranged in size in a range of a few hundred micrometers.

**Figure 4:**
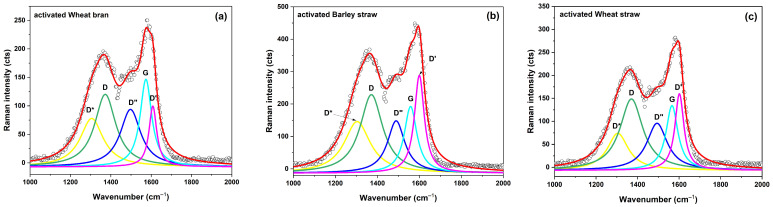
Raman spectra of activated wheat bran (**a**), barley straw (**b**), and wheat straw (**c**).

The Raman spectra of the RH, BS, WhS, and WhB samples, recorded in the spectral range of 1000–2000 cm^−1^, are presented in Figure 4. The spectra were deconvoluted using Lorentzian functions to accurately isolate the first-order modes: the D*, D, D″, G, and D′ bands. This deconvolution method allowed for a more precise analysis of the structural features and defects in the carbon materials, addressing the limitations of the traditional I(D)/I(G) ratio, which can be skewed by band overlap.

The G band, observed at 1583 cm^−1^, corresponds to the E2g optical phonon mode, characteristic of sp2-hybridized carbon atoms in graphite-like materials [19]. This band is a key indicator of the degree of crystallinity of and structural changes in carbon materials. The D band, located between 1330 and 1350 cm^−1^, is attributed to defects and disorders in the carbon lattice, as well as double-resonance processes near the K point of the Brillouin zone [19]. The D″ band, appearing in the 1500–1550 cm^−1^ range, is associated with the amorphous phase, with its intensity inversely proportional to crystallinity [20]. The D′ band at 1620 cm^−1^ corresponds to the disorder-induced phonon mode associated with crystal defects. The D* band, found between 1050 and 1200 cm^−1^, depends on the remaining oxygen-containing groups [21].

The degree of graphitization for each sample was calculated using the ratio of the area under the G peak (A(G)) to the total area of all peaks (A_total_), as defined by the following equation [22]:

(1) $$Gf=\frac{A(G)}{A_{total}}$$

The WhB sample exhibited the highest degree of graphitization at 12.3%, indicating superior crystallinity and fewer defects relative to the other samples. The WhS sample displayed a graphitization degree of 10.0% but with lower-intensity the D and G bands, suggesting a different structural organization. The BS sample showed the lowest graphitization degree of 6.8%, correlating with the low intensity of the D and G bands, indicative of a more amorphous structure.

### 2.2. Electrochemical Test

The electrochemical performance of activated-carbon-based supercapacitor electrodes can vary significantly depending on the biomass source used for their derivation. This variation is primarily due to the differences in the pore structure and specific surface area that arise during the carbonization and activation processes.

**Figure 5:**
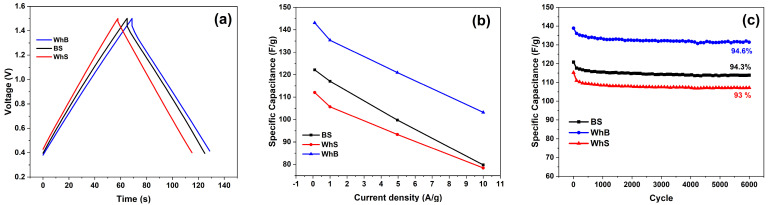
Electrochemical characteristics of supercapacitor electrodes obtained from barley straw, wheat straw, and wheat bran: (**a**) galvanostatic charge/discharge curves from 0.4 to 1.5 V at 1 A/g; (**b**) specific capacitances of the electrodes in a current range of 0.1–10 A/g; (**c**) cyclic stability test at 1 A/g from 0.4 to 1.5 V.

These factors influence the double-layer capacitance of the electrodes during constant current charge/discharge cycles. Figure 5a illustrates the galvanostatic charge/discharge curves at a current density of 1 A/g for the electrodes derived from different biomass sources.

The curves exhibit a typical behavior of electrical double-layer capacitor electrodes. No significant redox processes on the surface of the carbon electrodes in the given voltage ranges were observed.

Figure 5b presents the specific capacitances of the electrodes based on barley (BS), wheat straw (WhS), and wheat bran (WhB) as a function of charge/discharge current density. Among the tested materials, the electrodes based on wheat bran exhibited the highest specific capacitance, reaching up to 143 F/g at 0.1 A/g. This was followed by the electrodes based on barley and wheat straw, with specific capacitances of 122 F/g and 112 F/g, respectively.

As the charge/discharge current density increased to 10 A/g, the specific capacitance of the wheat-bran-based electrodes decreased to 103.2 F/g, maintaining 72.1% of its maximum value. In comparison, the wheat-straw-based electrodes showed a capacitance retention of 70%, while the performance of the barley-straw-based sample dropped to 65.3%.

Table 1 shows that the activated carbon from the wheat straw possessed the largest specific surface area, followed by barley and wheat bran. However, this order did not correlate with the specific capacitances, where the electrodes based on wheat bran exhibited the highest specific capacitance, followed by those based on barley and wheat straw. This discrepancy was likely due to the significant proportion of microscopic pores in these activated carbons, rendering the BET calculation method inaccurate.

Moreover, the higher graphitization degree of the active material (Figure 4) correlated with a greater retention of the specific capacitance as the charge/discharge current density increased. A high ratio of the intensities of the D and G peaks indicates a higher degree of crystalline structure destruction, leading to deteriorated charge conductivity.

Figure 5c gives information regarding the electrode cycling stability during tests conducted by charging/discharging in the potential range of 0.4–1.5 V at a constant current density of 1 A/g. The specific capacitances of all samples decreased slightly at the beginning of the test since initial current density was 0.1 A/g, and then maintained approximately 94% of the initial dataover 6000 cycles of charging and discharging at 1 A/g. At the same time, the Coulombic efficiency of all samples was more than 99%, which indicated an excellent charge and discharge reversibility.

**Figure 6:**
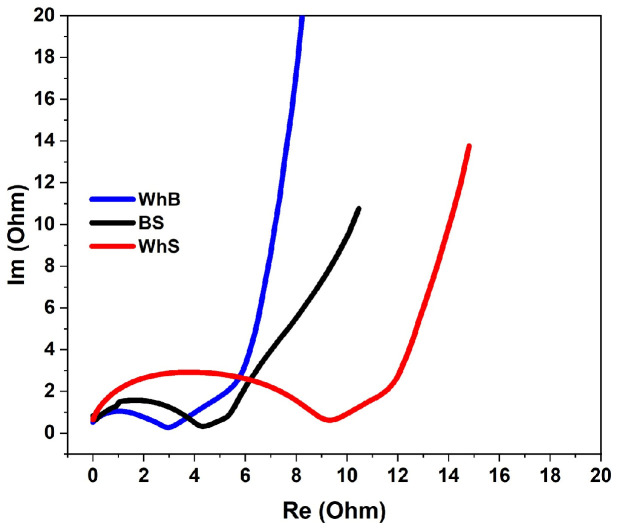
Nyquist plots of symmetrical coin cells with the electrodes based on activated carbons obtained from barley straw (BS), wheat straw (WhS), and wheat bran (WhB). The spectra were shifted to zero for clarity.

Figure 6 demonstrates the impedance spectra of the symmetrical coin cells that contained different activated carbons as the active material. All spectra contained semicircles in the high-frequency region, associated with charge transfer resistance (*R*_CT_), and the Warburg line in the intermediate-frequency region with a slope of about 45°, which is attributed to the diffusion capability of electrolyte ions. Among all the samples, the wheat-bran-based electrodes demonstrated the lowest *R*_CT_, reaching about 2.7 Ohm, while the *R*_CT_ for the BS- and WhS-based electrodes was 4.2 and 9.1 Ohm, respectively. Despite the difference in the Warburg impedance, the WhB-based electrodes showed the highest energy density of 33 Wh/kg at 122.6 W/kg, while that of the barley-straw-based sample was 29.66 Wh/kg at the same power density. The lowest energy density of 16.2 Wh/kg at 98 W/kg was demonstrated by the WhS sample. At the highest power density of 8 kW/h, the WhB sample showed an energy density of 10.1 Wh/kg, followed by the BS (6.54 Wh/kg) and WhS (5.7 Wh/kg) samples at a power density of 7.3 kW/h. For the power and energy density calculations, the mass of the active material was used.

These results underscore the importance of selecting appropriate biomass sources to produce activated carbon to optimize the electrochemical performance of supercapacitor electrodes. The differences in specific surface area, pore volume, and crystalline structure directly affect the charge storage capabilities and retention properties of the electrodes under varying current conditions. Additionally, by comparing FTIR spectra, specific surface areas, pore distributions, and electrochemical characteristics, the impact of biomass composition on the structural and electrochemical properties of activated carbon can be adequately elucidated.

**Figure 7:**
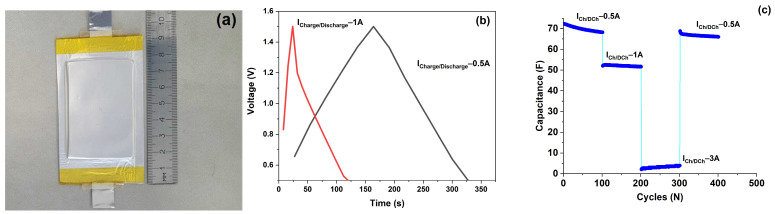
Image of the supercapacitor prototype (**a**), galvanostatic charge/discharge curves at different currents (**b**), C-rate performance (**c**).

Finally, two-side coated electrodes with a total areal mass loading of about 7 mg/cm^2^ were used in the pouch-cell-type supercapacitor prototyping. The wheat-bran-based activated carbon was used as the active electrode material. The electrode slurry content, separator, and electrolyte were the same as for the coin cells studied. The electrode area was 25 cm^2^ (4.3 × 5.8 cm). The prototype was stacked by eight electrodes with opposite current collectors (Figure 7a). The mass of the pouch cell supercapacitor was 4.16 g.

Figure 7b demonstrates the galvanostatic charge/discharge curves in a voltage range of 0.5–1.5 V, and the C-rate performance at a different current is shown in Figure 7c. The discharge capacitance of the prototype was 72 F at a discharge current of 0.5 A. Considering the total mass of the prototype, its energy density was estimated as 4.8 Wh/kg. The results support the scalability of the described laboratory electrode technology, which indicates that the proposed activated carbon is potentially suitable for large-scale production.

## 3. Materials and Methods

### 3.1. Materials

The following materials were used: aluminum foil (99.99%, 20 μm thick, Goodfellow); argon gas (99.99%, Ikhsan Technogas Ltd., Almaty, Kazakhstan); methane (99.9%, Ikhsan Technogas Ltd., Almaty, Kazakhstan); oxygen (99.5%, Ikhsan Technogas Ltd., Almaty, Kazakhstan); barley straw, wheat straw, and wheat bran (from Turkistan region, Kazakhstan); potassium hydroxide (KOH, purity not <85%, Sigma Aldrich); nitrogen gas (99.6% Ikhsan Technogas Ltd., Almaty, Kazakhstan); conductive carbon black (Super C45, MTI Corporation); polyvinylidene fluoride (PVDF, ≥99.5, MTI Corporation); N-methyl-2-pyrrolidinone (NMP, ≥99.0%, Sigma Aldrich).

### 3.2. Synthesis of Activated Carbon from Biomass

Activated carbon was obtained from barley straw (BS), wheat straw (WhS), and wheat bran (WhB) by pyrolysis (carbonization) and subsequent thermochemical activation. Before pyrolysis, the initial samples were cleaned and ground. The cleaning process included washing with hot water using household chemicals and rinsing in distilled water, followed by drying in a drying oven at a temperature of 110 °C for 12 h. Drying time may vary depending on the amount of wet substance and the type of drying oven used. The pyrolysis process was carried out at a temperature of 550 °C for 100 min in a vertical tube furnace in a nitrogen gas atmosphere at a flow rate of 150 SCCM. Thermochemical activation was carried out in a stainless-steel reactor (AISI321), where the carbonized mass was mixed with potassium hydroxide in a ratio of 1:4. Next, the reactor was heated to a temperature of 850 °C at a heating rate of 7 °C/min with a supply of gaseous nitrogen (150 SCCM) and a holding time at a temperature of 850 °C for 120 min. The cooling process during both pyrolysis and chemical activation was carried out in a natural way.

### 3.3. Cell Fabrication and Electrochemical Measurements

The electrochemical characteristics of the electrodes containing highly porous activated carbon derived from barley straw, wheat straw, and wheat bran were assessed using a Neware BTS workstation (https://newarebattery.com/, China).

In order to prepare the electrode slurry, polymer binder PVDF Solef 5130 (TMax, China) was dissolved in N-methyl pyrrolidone for 2 h at 60 °C under stirring. After complete dissolution of the PVDF, a pre-mixed sample of the active material and the conductive additive Super C45 (TMax, China) was added and stirred on an overhead mixer for 20 h to form a homogeneous slurry.

Next, the obtained electrode slurry was stirred on a vacuum mixer for 20 min to remove gaseous substances. After that, the slurry was applied to aluminum foil using the Doctor Blade technique (Gelon, China) and dried at 100 °C for 12 h until the solvent completely evaporated.

After complete drying, the electrode tape was calendared at a temperature of 100 °C with a compression ratio of 20%. Furthermore, the electrodes were laser-cut on discs with an area of 1.77 cm^2^ for 2032-coin cells.

The electrodes were weighed and vacuum-dried at a temperature of 120 °C for five hours to remove trace amounts of water and transferred to a glove box, where the coin cells were assembled in an argon atmosphere. 

Symmetric supercapacitor coin cells were assembled with an organic electrolyte based on acetonitrile (1M TEATFB in AN), employing Celgard 2500 (TMax, China) as a separator. Each electrode comprised 92 wt% of synthesized activated carbon as the active material, 3 wt% conductive additive, and 5 wt% binder. The total electrode areal mass loading was approximately 4.7 ± 0.7 mg/cm^2^. Specific capacitance was determined from galvanostatic charge/discharge curves in a voltage range of 0.4–1.5 V at a current density of 1–10 A/g.

The electrode capacitance C was calculated based on the galvanostatic charge/discharge curves of coin cells according to the following equation:

C = 2 × (I × ∆t)/∆V(2)

where I is the charge/discharge current, Δt is the charge/discharge time, ΔV is the voltage window. 

Gravimetric capacitance (F/g) was calculated by dividing the capacitance C by the mass fraction of the active material in the electrode layer.

### 3.4. Characterization of Obtained Samples

The carbon structures obtained on a catalytic nickel substrate were characterized using an NTEGRA Spectra Raman spectrometer (The Netherlands) operating at a wavelength of λ = 473 nm. Surface morphology was examined via scanning electron microscopy (SEM) using a JEOL JSM-6490LA instrument (Japan), with magnifications ranging from ×5 to ×300,000 and an accelerating voltage from 0.1 to 30 kV. An elemental analysis of the carbon structures was performed using energy-dispersive X-ray (EDX) on the same SEM instrument. The specific surface area of the activated carbon was determined by nitrogen adsorption–desorption isotherms at 77 K using a gas sorption analyzer BSD-660S (China).

## 4. Conclusions

In conclusion, this research highlights the viability of using waste biomass to produce activated carbons with desirable properties for supercapacitor applications. The FTIR and Raman spectroscopic analyses reveal the structural transformations and functional group changes during carbonization and activation, which are critical for understanding the performance of the resultant activated carbons. The electrochemical evaluations show that the source of the biomass significantly influences the specific capacitance and energy density of the supercapacitors. Among the tested materials, wheat-bran-derived activated carbon exhibited the highest specific capacitance and energy density, making it a promising candidate for high-performance energy storage devices. The specific capacitance of the electrodes based on wheat-bran-derived activated carbon reached 143 F/g at 0.1 A/g.

At the same time, the activated carbons studied in this work demonstrated high cycling stability, reaching about 94% of the initial capacitance over 6000 cycles at 1 A/g. 

These findings underscore the importance of selecting appropriate biomass sources and optimizing activation processes to enhance the electrochemical performance of supercapacitors. This study provides valuable insights into the sustainable production of advanced materials for energy storage, promoting the development of efficient and eco-friendly supercapacitors.

## Figures and Tables

**Table 1:** Specific surface area and total pore volume of the activated carbons.

Activated Carbon	SSA (BET Method), m^2^/g	Total Pore Volume, cm^3^/g
WhB	1866.24	1.42
BS	1970	1.39
WhS	2036	1.23

## Data Availability

The processed data required to reproduce the above findings are available on request.
